# Multiple liver metastases with synchronous gastric and transverse colon cancer diagnosed by gastric perforation successfully treated by SOX plus bevacizumab and completely resected by surgery: a case report

**DOI:** 10.1186/s40792-020-00808-x

**Published:** 2020-03-16

**Authors:** Ryu Matsumoto, Shinichiro Mori, Yoshiaki Kita, Hiroko Toda, Ken Sasaki, Takaaki Arigami, Daisuke Matsushita, Hiroshi Kurahara, Kosei Maemura, Shoji Natsugoe

**Affiliations:** Department of Digestive Surgery, Breast and Thyroid Surgery, Graduate School of Medical and Dental Sciences, Kagoshima University, 8-35-1 Sakuragaoka, Kagoshima-shi, Kagoshima, 890-8520 Japan

**Keywords:** Synchronous double cancer, Colon cancer, Gastric cancer, Liver metastasis, Chemotherapy, SOX, Bevacizumab

## Abstract

**Background:**

Synchronous double cancer of the colon and stomach accompanied by liver metastasis is rare. It is often difficult to determine an appropriate treatment strategy for multiple liver metastases of synchronous gastric cancer and colorectal cancer. Multidisciplinary treatment is required based on the progression and location of each tumor and chemotherapy for complete resection.

**Case presentation:**

A 57-year-old male who complained of acute abdominal pain and fever visited his local hospital. He underwent emergent surgery for peritonitis caused by a gastric perforation. The cytodiagnosis of ascites did not show any tumor cells. There was a liver metastasis in the lateral segment of the liver. We performed a primary closure of the defect and then applied an omentum flap. After surgery, the patient was diagnosed as having synchronous cStage IV transverse colon cancer with multiple liver metastases and cStage IIB gastric cancer. The [18F]-fluorodeoxyglucose (FDG) positron emission tomography/computed tomography (PET/CT) showed 18F-FDG uptake by the colon tumor and multiple liver metastases, but there was no uptake in the gastric tumor or lymph nodes. We retrospectively reevaluated the CT findings from a local hospital and detected a liver nodule in segment 2/3 (from 35 to 60 mm) and segment 6 (from 26 to 57 mm), and the tumors had dramatically grown in size in only 2 months. Because complete tumor resection would be difficult, S-1 and oxaliplatin (SOX) plus bevacizumab therapy was started to control tumor progression. After 20 courses of chemotherapy, the clinical diagnosis was ycStage IV transverse colon cancer and ycStage IIa gastric cancer. We planned a two-step procedure to completely resect the primary tumors and multiple liver metastases. We first performed a laparoscopic right-colon resection+D3 lymphadenectomy and open distal gastrectomy+D2 lymphadenectomy. The patient was discharged home on postoperative day 18. After 1 month, we performed open liver resection. The pathological findings showed that the transverse colon was ypT2 (MP) with grade 2 therapeutic effects and that there were no atypical cells in the gastric tumor and multiple liver nodules (pathological complete response).

**Conclusion:**

The SOX plus bevacizumab regimen could be an option for controlling tumor progression in synchronous double cancer of the colon and stomach with liver metastasis and led to the complete resection of such tumors.

## Background

Synchronous double cancer of the colon and stomach is relatively rare, especially when accompanied by liver metastasis. It is often difficult to determine an appropriate treatment strategy for multiple liver metastases of synchronous gastric cancer and colorectal cancer. Multidisciplinary treatment is required based on the progression and location of each tumor and chemotherapy for complete resection [1]. Recent developments in chemotherapy and the device of minimally invasive surgical procedures such as laparoscopic surgery enabled the successful complete resection of synchronous double cancer with multidisciplinary treatment [1, 2]. We experienced an important case of synchronous double cancer of the transverse colon and stomach accompanied by multiple liver metastases that was completely resected following S-1 and oxaliplatin (SOX) plus bevacizumab, and the pathological findings showed complete response in the gastric cancer and liver metastases.

## Case presentation

**Fig. 1:**
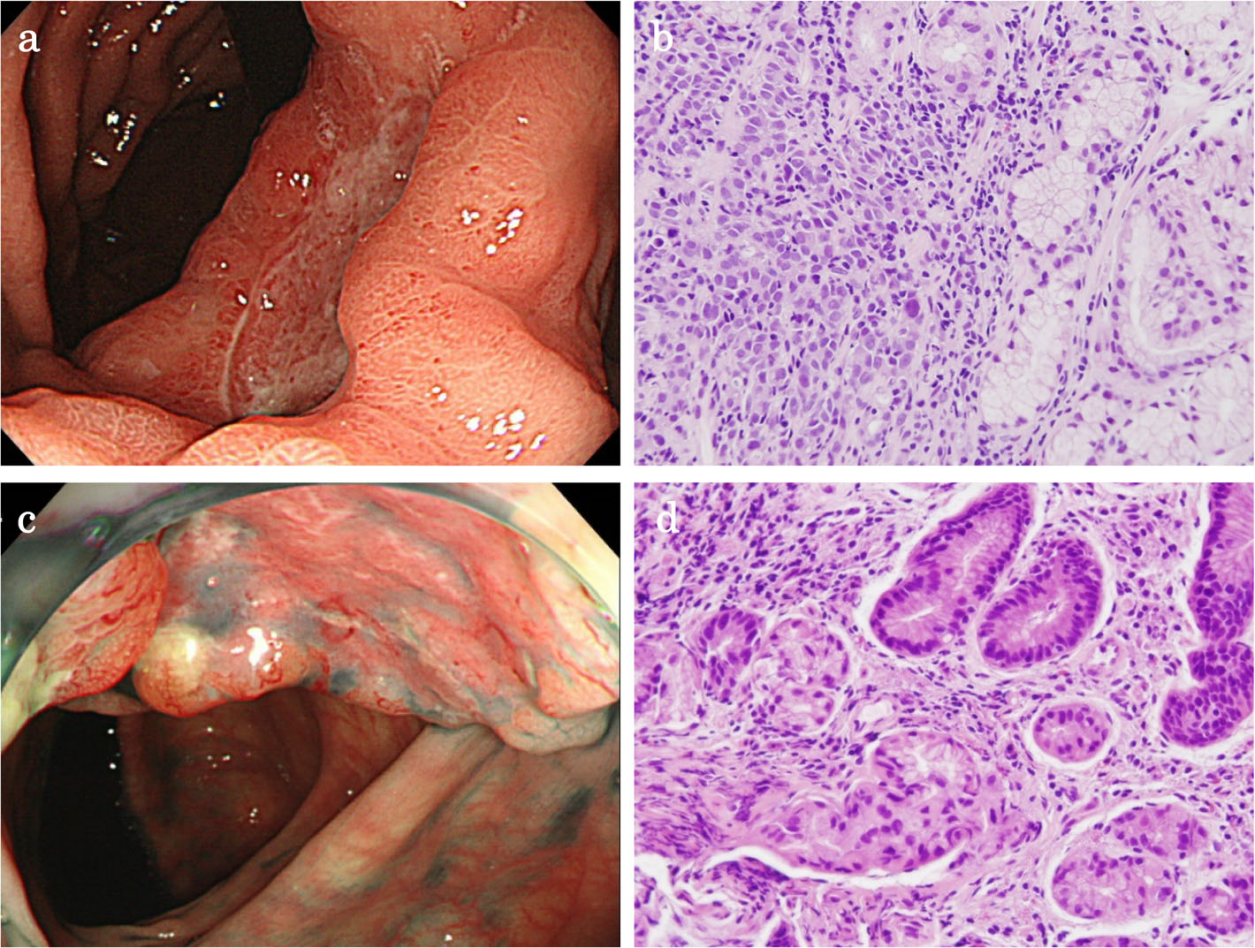
Imaging findings before chemotherapy. **a** Gastroscopy showed a type II tumor at the lesser curvature of the gastric body. **b** Pathological findings indicated poorly differentiated adenocarcinoma. **c** Colonoscopy showed a type II tumor with a diameter of 25 mm in the transverse colon. **d** Pathological findings indicated well-differentiated tubular adenocarcinoma

**Fig. 2:**
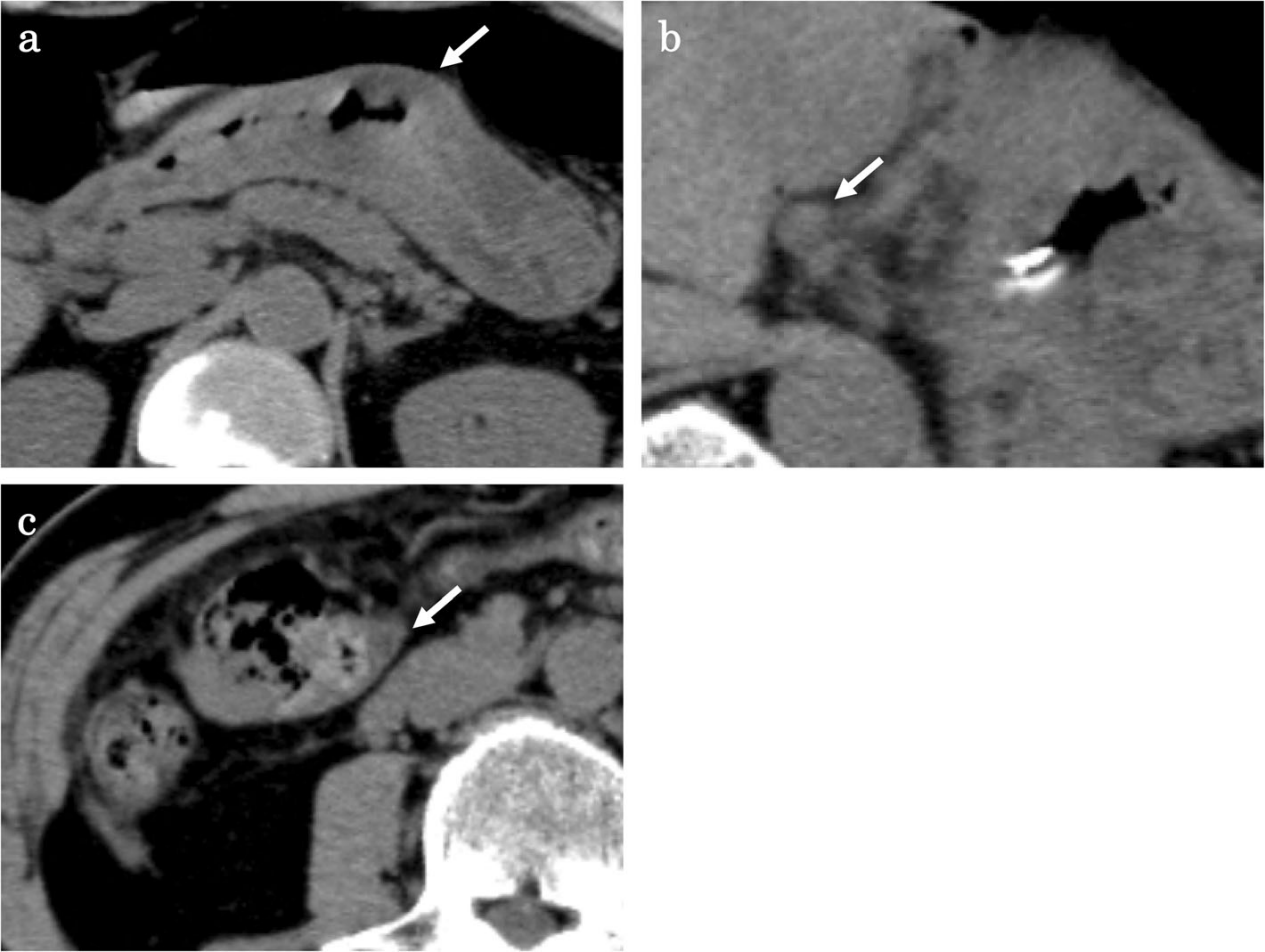
CT findings before chemotherapy. **a** CT scan showed irregular wall thickness at the gastric body (white arrow). **b** CT scan showed lymph node enlargement at station 3 with a size of 7 × 11 mm (white arrow). **c** CT scan showed irregular wall thickness in the transverse colon (white arrow)

**Fig. 3:**
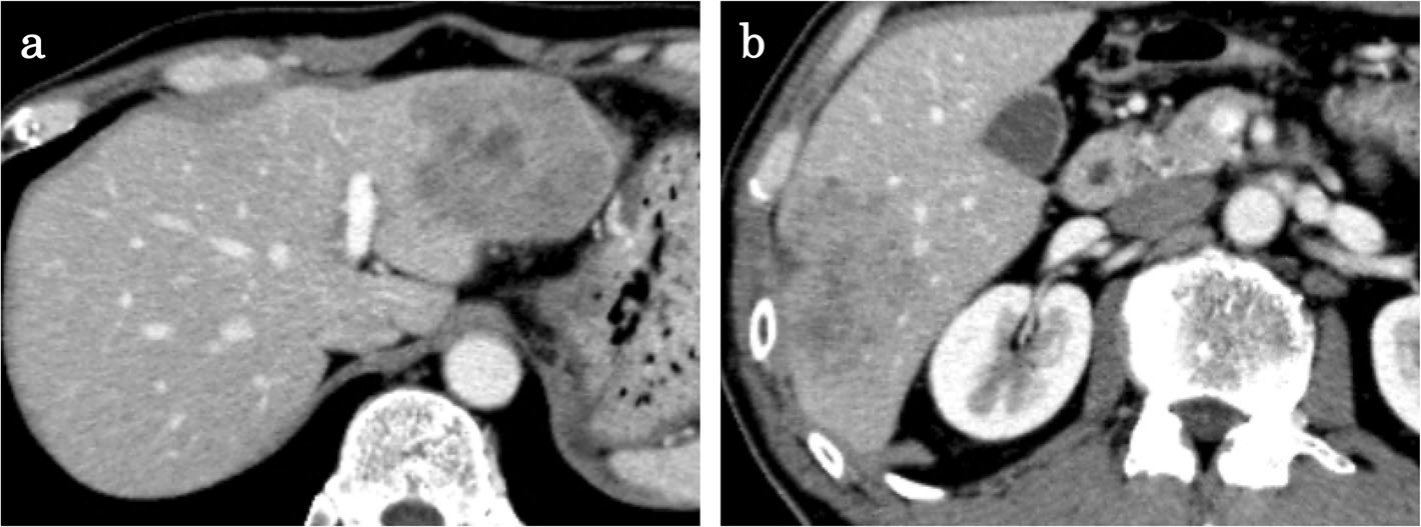
Liver nodules before chemotherapy. **a**, **b** Enhanced CT scan showed irregular low-density nodules in segment 2/3 (**a**, 60 mm in diameter) and segment 6 (**b**, 57 mm in diameter), which were suspected to be liver metastases

**Fig. 4:**
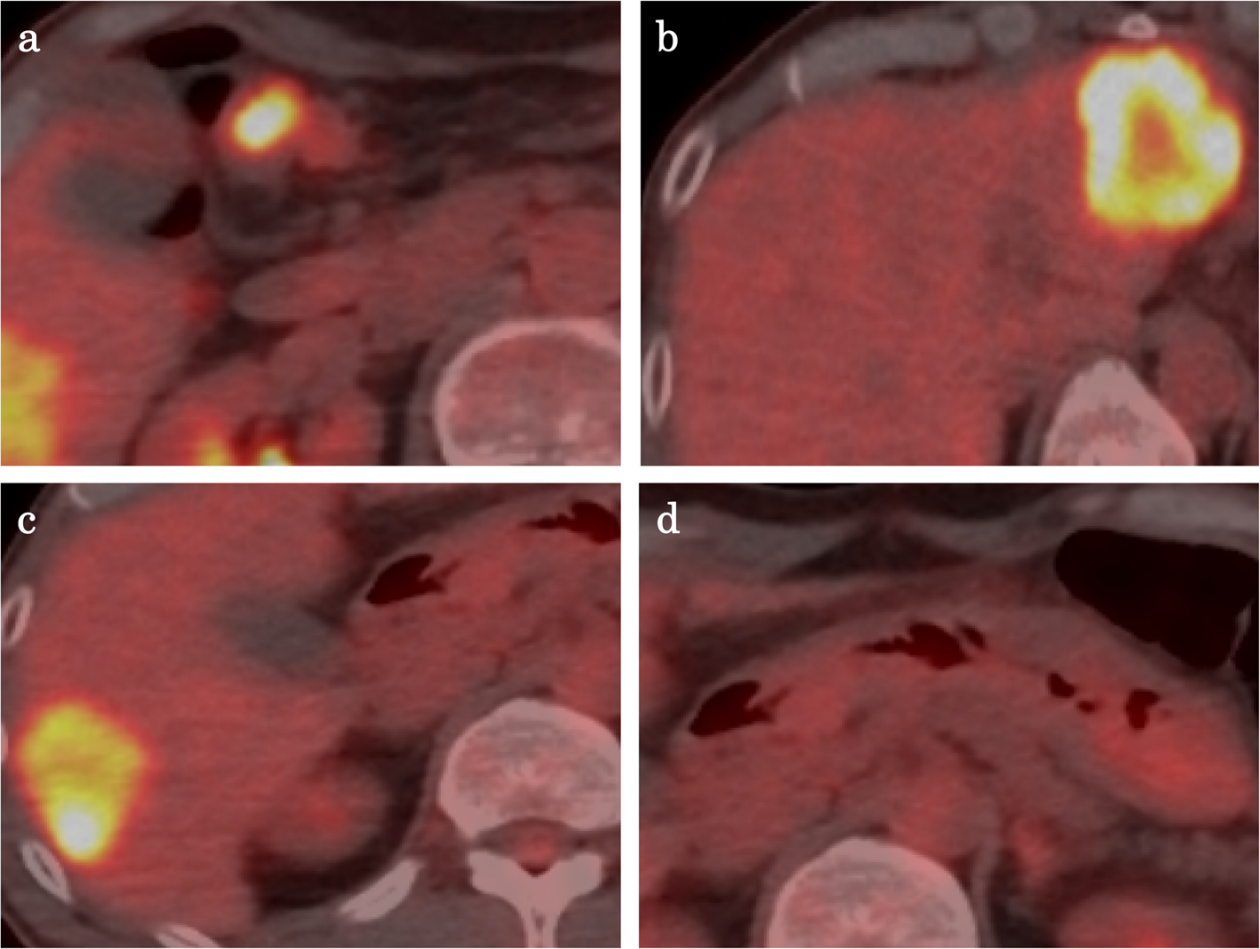
FDG PET/CT findings before chemotherapy. FDG PET/CT findings showed 18F-FDG uptake by the transverse colon cancer (**a**, SUVmax of 14.4), multiple liver metastasis in segment 2/3 (**b**, no SUVmax measurement) and in segment 6 (**c**, SUVmax of 11.3), but there was no uptake in the gastric body (**d**).

**Fig. 5:**
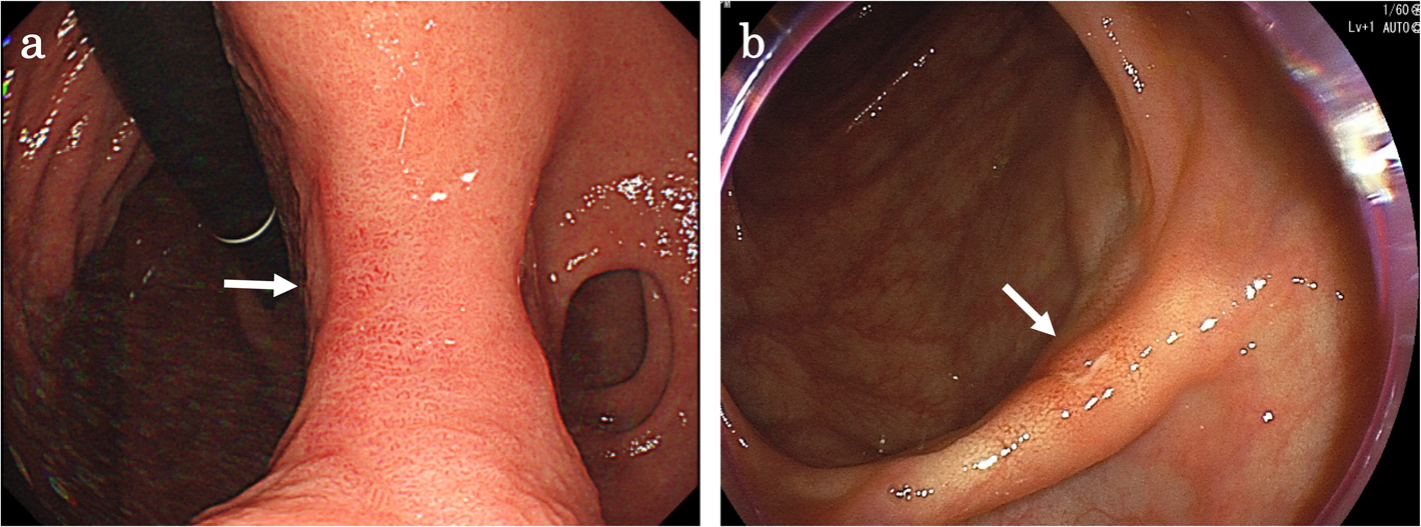
Endoscopy findings after chemotherapy. **a** Gastroscopy and colonoscopy showed scarring at the lesser curvature (**a**) and transverse colon (**b**) (white arrow)

**Fig. 6:**
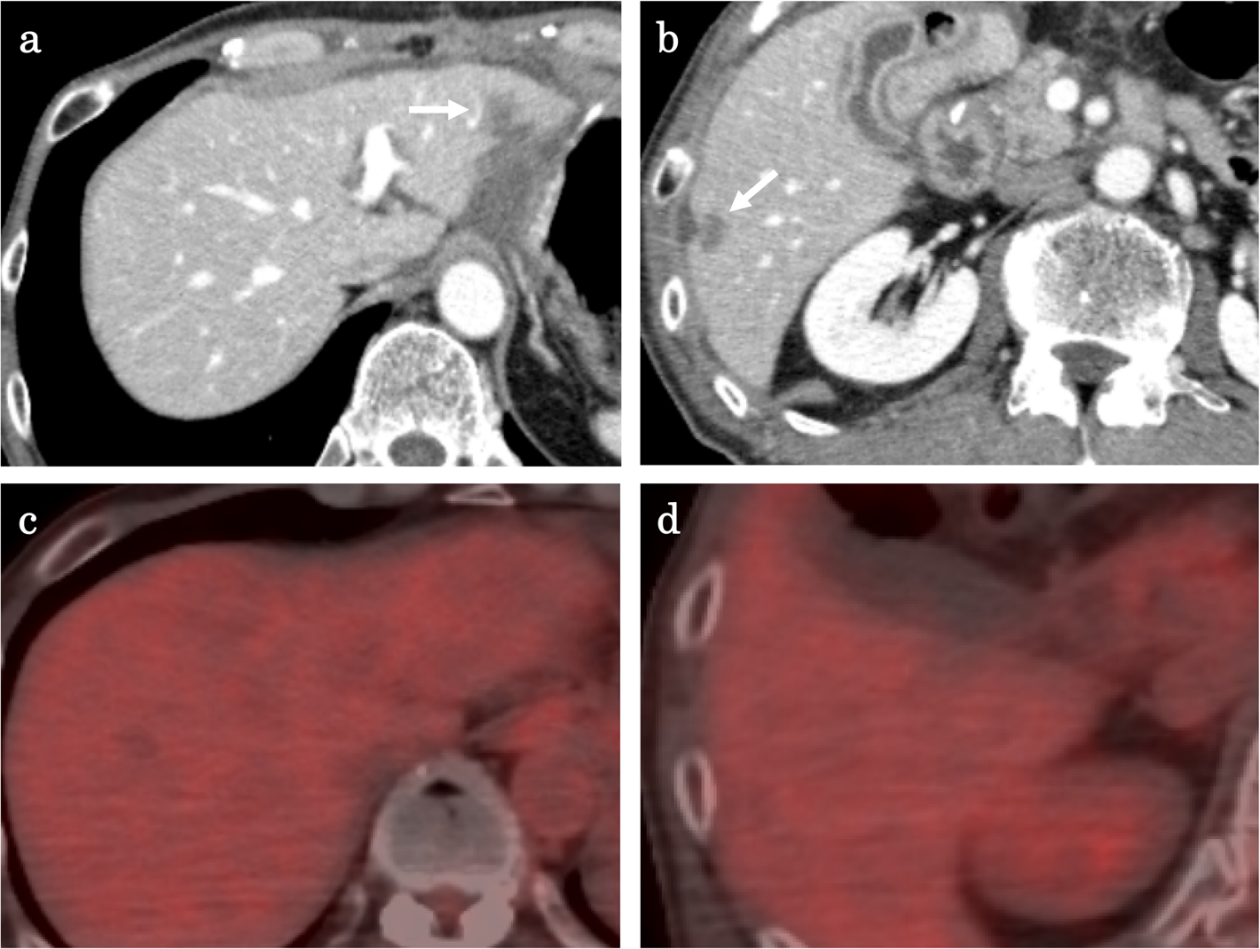
Liver nodules after chemotherapy. The hepatic nodules in segment 2/3 and segment 6 had dramatically reduced in size to 14 mm in diameter (**a**) and 15 mm in diameter (**b**), respectively (white arrow). **c**, **d** Neither tumor showed 18F-FDG uptake

A 57-year-old male who had no family history of cancer complained of acute abdominal pain and fever and visited his local hospital. He was diagnosed with gastric perforation and referred to our hospital. We performed emergent surgery for peritonitis caused by a gastric perforation. We evaluated the abdominal cavity laparoscopically and found some cloudy ascites in the abdomen and a pin hole perforation at the anterior wall of the gastric antrum. Because the gastric wall around the perforation was thick and did not have any serous changes, it was difficult to assess whether the cause of the perforation was tumor related. The cytodiagnosis of ascites did not show any tumor cells. There was also a white nodule in the lateral segment of the liver, which was suspected to be a metastatic liver tumor. We performed a primary closure of the defect, applied an omentum flap and washed the abdominal cavity with 10 l of normal saline. The operation time was 120 min, and the volume of blood loss was 10 ml. The postoperative course was uneventful. We performed upper and lower endoscopy, which showed type II tumors in the gastric body (poorly differentiated adenocarcinoma, HER2 score 2+) (Fig. 1a, b) and transverse colon (well-differentiated tubular adenocarcinoma, RAS mutation) (Fig. 1c, d), and we considered that the gastric perforation was related to the presence of advanced gastric tumors. Furthermore, the CT showed irregular wall thickness with ulcers in the gastric body, which were suspected to be gastric cancer with lymph node metastases at station no. 3, irregular wall thickness of the transverse colon (Fig. 2a–c), which was suspected to be colon cancer, and nodules in liver segments 2/3 (60 mm) and in segment 6 (57 mm) (Fig. 3a, b), which were suspected to be liver metastases. We retrospectively reevaluated the CT findings from a local hospital and detected liver nodules in segment 2/3 (35 mm) and segment 6 (26 mm), and the tumors had dramatically grown in size in only 2 months. [^18^F]-fluorodeoxyglucose (FDG) positron emission tomography (PET)/CT showed colon cancer (maximum standardized uptake value (SUVmax) 14.4) and multiple liver metastases (S2/S3, unclear SUVmax; S6, SUVmax 11.3), but 18F-FDG uptake was not found in the gastric tumor and lymph nodes (Fig. 4a–d). The serum blood tests showed normal tumor marker levels (carcinoembryonic antigen, 4.6 mg/dl; carbohydrate antigen 19-9, 20.7 mg/dl) and normal liver function. We diagnosed the patient with synchronous cStage IV (cT3cN0cM1) transverse colon cancer with multiple liver metastases and cStage IIB (cT3cN1cM0) gastric cancer according to the 7th edition TNM classification [3]. Because of the gastric cancer perforation and the dramatic enlargement of the liver metastasis, we believed that a complete resection of the tumors would be difficult, and that chemotherapy treatment would be important to control tumor progression. We chose SOX plus bevacizumab therapy (on day 1 of each 3-week cycle, 7.5 mg/kg intravenous infusion of bevacizumab and 130 mg/m^2^ intravenous infusion of oxaliplatin were administered along with a dose of S-1 60 mg/m^2^ twice a day starting after dinner on day 1 until after breakfast on day 15, followed by a 7-day break). After 10 courses, the patient presented with oxaliplatin-induced peripheral neuropathy [4], and another 10 courses were administered without oxaliplatin. An endoscopic examination did not detect gastric cancer (Fig. 5a) or transverse colon cancer (Fig. 5b). The liver metastases were dramatically reduced in size to 14 mm in segment 2/3 and 15 mm in segment 6 (Fig. 6a, b), and the FDG PET/CT findings showed no 18F-FDG uptake in any of the tumors (Fig. 6c, d). The serum blood tests showed the elevated tumor marker levels (carcinoembryonic antigen, 12.6 mg/dl; carbohydrate antigen 19-9, 43.1 mg/dl). After chemotherapy, the clinical diagnoses were ycStage IV (ycT3ycN0ycM1) transverse colon cancer and ycStage IIa (ycT3ycN0ycM0) gastric cancer. We planned a two-step procedure to completely resect the primary tumors and multiple liver metastases. We first performed resection of the colon and gastric tumors. Intraoperatively, there were broad adhesions between the omentum and the abdominal wall. The cytodiagnosis of the ascites showed no atypical cells. A small white nodule in segment 6 was detected. No signs of a “blue liver” were detected. We first laparoscopically performed resection of the transverse colon cancer in the right-side colon along with D3 lymphadenectomy. Then, we performed open distal gastrectomy for the gastric tumor with D2 lymphadenectomy (Roux-en-Y anastomosis). The operation time was 605 min, and the volume of blood loss was 840 ml. The patient was discharged home on postoperative day 18 and had an uneventful postoperative course. After 1 month, we performed liver resection for the multiple liver metastases. Intraoperatively, we detected tumors in segment 6 on the liver surface, and with sonography, we detected a tumor in segment 3, which was very close to the umbilical portion of the portal vein. We performed open lateral segment resection and partial resection at segment 3. The operation time was 402 min, and the volume of blood loss was 420 ml. The patient was discharged home on postoperative day 12 without any remarkable complications. The pathological findings showed ypT2 (MP) tumors with grade 2 therapeutic effects in the transverse colon and no residual gastric tumor and multiple liver nodules (pathological complete response).

## Discussion

Approximately 2 to 7% of patients with colorectal cancer have one or more synchronous cancers at the time of the initial diagnosis [5]. The major sites of the other primary cancers are the stomach (43.8%) and lung (15.3%) in males and the mammary glands (32.4%) and uterus (25.5%) in females [6]. The treatment strategy should be planned according to the stage of each individual tumor. We mostly considered colon cancer to be the primary disease because colon cancer is usually more likely to metastasize to the liver than gastric cancer. The FDG PET/CT findings showed 18F-FDG uptake in only the colon tumor and liver metastasis. Furthermore, poorly differentiated gastric cancer often shows low 18F-FDG uptake [7, 8].

In our case, the liver metastases dramatically enlarged in the 2 months since the gastric perforation that required emergent surgery. Nojiri et al. reported that the invasive operation itself affects the presence of E-cadherin in the vascular endothelium, which promotes metastasis [9]. Furthermore, there were several reports that surgical stress and primary tumor resection could be related to metastasis after the operation [10, 11] and the IL-6 and growth hormone were also reported to be closely related to metastases of colorectal cancer [12]. We considered that the peritonitis caused by a gastric perforation and surgical stress promoted liver metastasis. The ascites was negative for tumor cells; however, the gastric perforation may have disseminated the tumor cells into the abdomen. For these reasons, we first selected chemotherapy with SOX plus bevacizumab to shrink both tumors and to control the micrometastases. SOX has been shown to be effective for gastric cancer [13, 14], and SOX plus bevacizumab has been shown to be effective for metastatic colorectal cancer [15]. Therefore, we selected the SOX plus bevacizumab regimen for this patient. The timing of conversion therapy is a crucial point in this case. The patient underwent enhanced CT examination every 2 months to evaluate the possibility of conversion surgery. The size of multiple liver metastases did not dramatically change in the first 2 months after the induction of SOX plus bevacizumab. Therefore, we continued the chemotherapy. We also concern about dissemination due to perforation from gastric cancer. It did not appear during chemotherapy. However, the tumor markers were gradually elevated; therefore, we considered that the patient was refractory to this regimen and planned the two-step conversion surgery.

Preoperative chemotherapy was expected to be more effective than similar postoperative treatment strategies for esophageal, gastric, and rectal cancers because it can effectively eradicate micrometastases and reduce the risk of incomplete excision and tumor cell shedding during surgery [16]. Moreover, the FOxTROT Collaborative Group showed that the resection rate for cT3/4 locally invaded colon cancer was significantly superior compared with that for other stages of cancer [16]. However, the data on the long-term oncological outcomes of preoperative chemotherapy for colon cancer are insufficient, and the regression of locally advanced colon cancer has been reported [17]. In this case, we controlled the progression of the primary tumors and liver metastases via the SOX plus bevacizumab regimen and performed complete resection. Furthermore, the gastric cancer and multiple liver metastases achieve a pathological compete response. The SOX plus bevacizumab regimen could be an option for synchronous double cancer of the colon and stomach with liver metastasis.

We administered capecitabine orally at a dose of 1000 mg/m^2^ twice daily as adjuvant therapy for 6 months. We firstly considered the S-1 regimen for adjuvant chemotherapy because the neoadjuvant chemotherapy including S-1 regimen was very effective and the tumors disappeared in gastric cancer and liver metastases. On the other hand, because the tumor markers such as CEA and CA19-9 were elevated and the refractory of S-1 regimen was suspected, we performed surgical resection. Therefore, we considered that the patient was refractory to S-1 regimen. We finally administered capecitabine to change the regimen. The capecitabine and oxaliplatin regimen is effective for both colon cancer and gastric cancer as adjuvant therapy [18, 19]. Although the patient’s oxaliplatin-related peripheral neuropathy resolved to a grade 1 reaction, we only administered capecitabine because he refused oxaliplatin. The patient did not have any complications during adjuvant therapy. The patient is currently alive 9 months after the first surgery and has not experienced tumor recurrence.

## Conclusions

In conclusion, we applied multidisciplinary treatment and performed a two-step procedure to completely resect the synchronous double cancer of the transverse colon and stomach accompanied by liver metastasis. The SOX plus bevacizumab regimen was a very effective treatment to control tumor progression for synchronous colon and gastric cancer with liver metastasis.

## Data Availability

Ethical approval was obtained from the Ethics Committee of Kagoshima University Hospital.

## Abbreviations

CT: Computed tomography
FDG PET: Fludeoxyglucose positron emission tomography
MRI: Magnetic resonance imaging
SOX: S-1 plus oxaliplatin
SUV: Standardized uptake value
